# Reasons and impacts of alcohol use disorder among ethnic minority young adults: a descriptive phenomenological study

**DOI:** 10.3389/fpubh.2025.1560092

**Published:** 2025-06-19

**Authors:** Getaneh Mulualem Belay, Mak Yim Wah, Katherine Ka Wai Lam, Qi Liu, Mao Ting, Chen Xi, Funa Yang, Cynthia Sau Ting Wu, Ka Yan Ho

**Affiliations:** School of Nursing, Hong Kong Polytechnic University, Hong Kong, China

**Keywords:** alcohol use disorder, ethnic minority, impacts, reasons, young adults

## Abstract

**Introduction:**

Alcohol use disorder (AUD) is prevalent among ethnic minority young adults. However, there is limited evidence exploring its health impact and contributing factors in ethnically marginalized young adults. Therefore, this study examined the reasons for developing AUD and its effect on the daily lives of ethnic minority young adults.

**Method:**

A descriptive phenomenological study was conducted from March 1 to June 30, 2023. Participants were recruited through referral using the exponential snowball sampling technique. A total of 22 ethnic minority young adults were interviewed using a semi-structured interview guide. The sample size was determined based on data saturation. All interviews were audio-recorded and transcribed verbatim in English. Then, the data was analyzed using Colaizzi’s method.

**Results:**

This study suggested that the reasons for developing AUD among ethnic minority young adults were (1) culture and family, (2) hedonistic motives, (3) curiosity, (4) low-risk perception, (5) coping motives, (6) social influences, and (7) subjective cravings. Moreover, this study indicated that ineffective daily performance, emotional turmoil, financial constraints, and social relationship issues were the impacts of AUD on this segment of the population.

**Conclusion:**

The findings highlighted the multifaceted reasons contributing to AUD among ethnic minority young adults, including coping, low-risk perception, and influences from culture, family, and peers. The findings also showed how AUD severely affected their daily lives. Therefore, future interventional studies should consider cultural, family, social, and psychological aspects.

## 1 Introduction

Alcohol use disorder (AUD) is a medical problem characterized by the inability to reduce or limit alcohol consumption despite adverse social, occupational, or health consequences (1). It is a common substance use disorder among young adults (2, 3). Early initiation of alcohol use, particularly before age 15, significantly increases the risk of AUD, with studies indicating a fourfold risk compared to starting after age 21 (4). Regarding its impact, previous studies have identified several consequences of AUD, including poor academic performance, sexual assault, injuries, car accidents, and fatalities (5, 6). Notably, the World Health Organization (7) reported that young adults aged 20 to 39 represent 13.5% of the estimated 3 million alcohol-related deaths.

AUD is prevalent in ethnic minority young adults (8–10). In 2014, a study conducted in the US on ethnic minorities revealed that the prevalence of AUD in Puerto Ricans was 3.6, 2.5% in Mexican Americans, 1.6% in Hispanics, and 0.6% in Cuban Americans (11). An additional US study in 2011 on ethnic minority young adults revealed a significant variation in AUD prevalence, that is, 2.6% in American Indian, 1.0% in Asian American, and 0.8% in Black American (12). The Substance Abuse and Mental Health Administration (SAMHSA) also reported that the prevalence of AUD was 12.7% in American Indians, 7.1% in American Asians, and 6.4% in Black Americans (13). However, the reason for this variation among ethnic minority young adults was unclear.

Currently, the number of Hong Kong ethnic minority groups is increasing, which accounts for about 8.4% of Hong Kong’s population during the census of 2021 (14). The demographic distribution shows Filipino as the largest group (32%), followed by Indonesian (23%), white (10%), Indian (7%), Pakistani (4%), Nepalese (5%), Japanese (2%), Thai (2%), other Asian (4%), and others (11%) (14). However, investigations revealed that ethnic minorities face significant challenges (15). For instance, many of them encountered discrimination, acculturation stress, marginalization, low self-esteem, and poor social integration (15–18). Ethnic minorities are considered underprivileged groups with low socioeconomic status (19–21). Despite a substantial governmental effort, their health remained a major concern (22). Furthermore, previous studies suggested that discrimination, poor family supervision, language barriers, social influences, acculturation, and culturally specific values and beliefs were reasons that significantly increased AUD prevalence (16, 23, 24). For instance, discrimination contributed to a 1.5-fold higher risk of mild AUD, a 1.6-fold higher risk of moderate AUD, and a 2.3-fold higher risk of severe AUD (25).

A considerable number of studies examined the issue of AUD in ethnic minorities. However, most studies adopted a quantitative approach and focused on determining AUD prevalence in different ethnic minorities and overlooked the reasons for developing AUD and its impact on their day-to-day activities (26, 27). This indicates that less attention has been paid to how AUD affects the daily lives of ethnic minority young adults. To date, there is limited qualitative evidence about how and why people initiate and maintain alcohol use and eventually develop AUD, particularly in ethnic minority young adults. Besides, there is a lack of qualitative studies summarizing how the daily lives of ethnic minority young adults are affected by AUD.

To shed light on the gap in existing literature, the current study explored the reasons for developing AUD and its impact on daily lives among ethnic minority young adults.

## 2 Methods and materials

### 2.1 Study design

A descriptive phenomenological qualitative study was conducted from March 15 to June 30, 2023. This approach helped researchers understand meaning, lived experiences, perceptions, and reasons behind the AUD phenomenon at the individual level (28, 29).

### 2.2 Study setting

This study was conducted in Hong Kong, a Chinese special administrative region located in the east of the Pearl River Delta. Hong Kong is a place where various ethnic minority groups live. It has approximately 7.5 million people with a land area of 1,104 square kilometers, of which about 619,552 (8.4%) are ethnic minorities (14). According to the census in 2021, the majority of ethnic minorities in Hong Kong were Filipino at 201,288 (32%), followed by Indonesian at 142,065 (23%), white at 61,579 (10%), Indian at 42,569 (7%), Pakistani at 24,385 (4%), Nepalese at 29,701 (5%), Japanese at 10,291 (2%), Thai at 12,972 (2%), other Asian at 24,580 (4%), and others at 70,122 (11%).

### 2.3 Study participant selection

**Figure 1:**
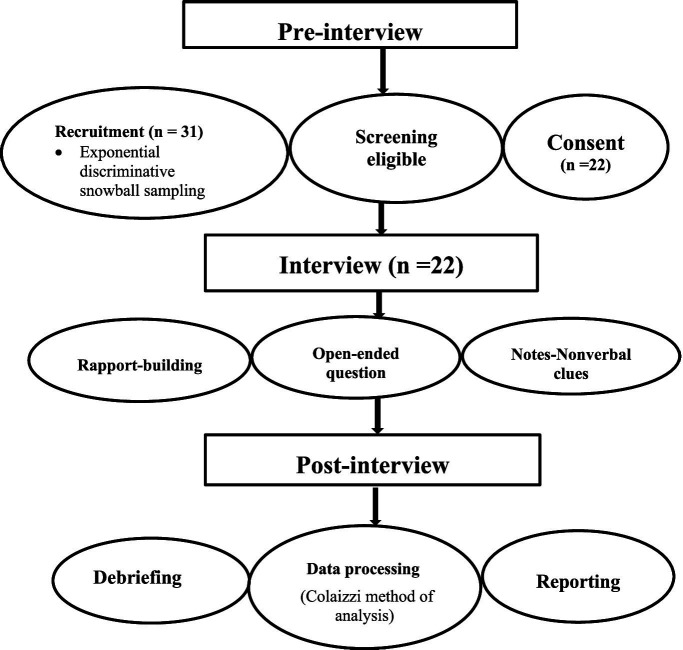
Schematic presentation of recruitment and interview procedures.

An exponential discriminative snowball non-probability sampling technique was employed to recruit participants (30). In exponential discriminative snowball sampling, the first subject recruited to the sample provides multiple referrals. Each new referral provided more potential participants. An African center, a for-profit company where Africans gathered, recommended the first participant for recruitment. Then, additional potential participants were recruited until data saturation. The data saturation was obtained upon interviewing a total of 22 respondents (Figure 1).

### 2.4 Eligibility criteria

The following criteria were considered for the study: (1) Hong Kong ethnic minority young adults who have resided in Hong Kong for at least six months and above because those participants drinking for less than 6 months may have fewer experiences of AUD in Hong Kong; (2) they must hear, read, speak, and understand conversations in English; (3) they have been legal residents of Hong Kong, with ages ranging from 17 to 30; (4) they had to participate voluntarily; and (5) they must have AUD by the self-assessment under the criteria defined by the Diagnostic and Statistical Manual of Mental Disorders, Fifth Edition (DSM-5) (31). Specifically, participants were considered to have AUD if they had at least two of the 11 symptoms outlined in the DSM-5 (31). However, apart from AUD, the participants must have no psychiatric or other cognitive problems.

### 2.5 Data collection

Each participant first spent around 10 min completing a sociodemographic questionnaire, the Alcohol Use Disorder Identification Test (AUDIT) (32), and the DSM-5 criteria. AUDIT is a screening tool that was initially used to identify risky drinkers. The total AUDIT scores range from 0 to 40, with a score of 8–15, 16–19, and 20 or above suggesting harmful or hazardous drinking, harmful, mild or moderate alcohol dependence, and severe dependence, respectively (32). Participants scoring >8, which indicates elevated risk of AUD, were further evaluated for AUD using self-administered DSM-5 criteria. Those meeting two or more DSM-5 criteria were included for qualitative interviews and invited to join a face-to-face, one-to-one, semi-structured interview with an audio recording. The recruitment was done in the *Tsim Sha Tsui*, *Kowloon* Chung King mansion. Tsim Sha Tsui (TST), a high-density commercial and entertainment district in Kowloon, Hong Kong, was selected for its geographical accessibility to participants (33). This location offers convenient access to our study population due to its high concentration of bars, and many ethnic minorities reside there, which provides a representative sample for our interviewees.

The interview was conducted by one interviewer who is a male registered nurse and a PhD candidate. The interviewer had no prior connection with the participants, but he had established a rapport with them before the interview. A semi-structured interview guide was used during the interview process. The interview guide included non-directive, open-ended, and adaptable interview questions developed by two senior qualitative researchers.

Each interview lasted between 10 and 30 min, and the time and date were chosen based on the participants’ preferences. Four primary topics were covered in the interview guide: (1) Their experiences with AUD; (2) How they started drinking; (3) Reasons for developing AUD; and (4) How AUD affects their lives (Supplementary File 1). All the interviews were audio-recorded. The interviewer documented all nonverbal cues during the interview. Finally, as a reward, participants who completed the interview received $30 to compensate for their time and travel expenses.

### 2.6 Data analysis

The demographic characteristics of the participants were summarized using descriptive statistics. The audio-recorded data were then transcribed into English verbatim for further analysis. All qualitative data were coded, and a table was used to describe the coded tree. Authors continuously cross-checked the audio recordings, transcriptions, and initial codes with the corresponding developed themes to (1) ensure the codes and themes were accurate and representative and (2) confirm the conceptual consistency between raw data and developed themes.

Colaizzi’s method of analysis has been used by the authors (34, 35). It is a rigorous method of analysis that employs a systematic, step-by-step process to examine participants’ narratives (36). This approach ensures an in-depth exploration of participants’ lived experiences. Particularly, the following steps were conducted throughout the analysis: (1) The authors became familiar with the data by reading and re-reading the transcripts and listening to the audio recordings numerous times until they fully comprehended its general meaning; (2) the authors systematically generated initial codes using the key features of the data across the entire data set; (3) themes were searched by collating the initial codes into the potential themes; (4) the developed themes were reviewed to see if they represented the data; (5) defining and naming themes; (6) finally, the results were reported per the Consolidated Criteria for Reporting Qualitative Research (COREQ) (37).

### 2.7 Research rigor

The lived experiences of AUD in ethnic minority young adults have been explicitly stated in the method and result sections to ensure the transferability of the findings. To ascertain reliability and credibility, the same interviewer has conducted interviews with each participant. The participants were instructed to express their opinions freely throughout the interview, and the interview was conducted in a quiet place to avoid disruptions and interruptions. Field notes were taken during the interview that included the location, date, and time, as well as any difficulties encountered. Themes and codes were continually compared to the existing data to determine whether they accurately reflect the participants’ actual experiences with AUD. Additionally, the research team has applied bracketing, in which a researcher suspends or holds in abeyance his or her presuppositions, biases, assumptions, theories, or previous experiences to see and describe the phenomenon (38). This bracketing was implemented throughout the pre-interview, interview, and post-interview phases. Prior to the interview, all team members explicitly documented assumptions in a reflexive memo. During interviews, the interviewer applied a semi-structured protocol with open-ended questions to avoid leading participants. The participants’ emotional reactions and contradictory assumptions, and the time and date of the interviews, were recorded in field notes. Following the interviews, two authors independently coded transcripts to minimize bias and then engaged in discussion to reach consensus to reconcile the discrepancies with the prespecified memos. To control the bracketing process, authors maintained a comprehensive audit trail through reflexive notes (39). The accuracy of the findings was also verified by professionals who have not been involved in the analysis. Any discrepancies were clarified through discussion and a second look at the analysis (Supplementary Table 1).

### 2.8 Ethical considerations

The Institutional Review Board (IRB) of the Hong Kong Polytechnic University granted ethical approval (reference number: HSEARS20230109001). Each participant was asked for their informed consent before the interview. All data gathered through face-to-face interviews with the participants was kept confidential in a non-identifying way. The confidentiality and privacy of the data were maintained throughout the research process.

## 3 Result

### 3.1 Socio-demographic characteristics

A total of 31 ethnic minority young adults with a history of drinking were referred by friends. Of these, 25 had AUD, and 22 agreed to participate. The average age of participants was 24.7 years (*SD* = 4.0). Most participants were female (68.2%), Catholic (59.1%), Filipino (50%), and single (77.3%). Over one-third (36.4%) had resided in Hong Kong for 5 to 10 years, and 40.9% had post-secondary education and were working as domestic helpers. The duration of participants’ drinking ranged from 1 to 15 years, with a median duration of 8 years (interquartile range: 5.5–11) (Table 1).

**Table 1:** Socio-demographic characteristics of ethnic minority young adult participants in Hong Kong (*n* = 22), 2023.

Variables	Number (*n*)	Percentage (%)
Sex		
Male	7	31.8
Female	15	68.2
Religion		
Catholic	13	59.1
Muslim	7	31.8
Christian	2	9.0
Living in Hong Kong		
<5 years	6	27.3
5–10 years	8	36.4
10–15 years	6	27.3
≥ 15 years	2	9.1
Ethnicity		
Filipino	11	50
Pakistan	1	4.5
African	10	45.5
Marital status		
Single	17	77.3
Married	3	13.6
others	2	9.1
Occupation		
Sale worker	1	4.5
Homemaker	4	18.2
Daily laborer	2	9.1
Craftsperson	1	4.5
Student	5	22.7
Domestic helper	9	40.9
Educational status		
Below primary	1	4.5
Junior secondary education	1	4.5
Senior Secondary	11	50
Post-secondary	9	40.9
Employment status		
Self-employed	5	22.7
Unemployed	3	13.6
Full-time employee	2	9.1
Part-time employee	12	54.5
Living arrangements		
Living alone	9	40.9
Living with parents	6	27.3
Living with relatives	1	4.5
Living with non-relatives	6	27.3
Duration of drinking in years		
Median (IQR)	8 (5.5–11)	

### 3.2 Reasons for developing AUD

#### 3.2.1 Theme: culture and family

Some participants (*n* = 4) reported that their culture and family had an impact on their initial engagement with various drinking options. Even though some parents were against their children’s drinking, a few participants (*n* = 3) stated that they were encouraged and invited to drink with their families. For example, one African participant indicated that he started to drink alcohol because his grandfather invited all family members, including children of all ages, to whiskey beverages. During the interview, he said, *“When I was young, my grandfather would call all of us because, in my culture, it’s a tradition that the grandfather has to share with all the grandchildren; it’s tradition. So, when I was young, whenever my grandfather had a bottle of whiskey, he had to call everyone to come, even if you were 10 years old, 12 years old, or 11 years old. You have to take a shot. So, that’s how I started drinking alcohol”* (29-year-old male African). Likewise, another participant from Africa said that invitations to family-related events were the main reason for his drinking and developing AUD, as indicated in his interview, *“What happened was a family event, and then my uncle asked me to try”* (21-year-old male African).

#### 3.2.2 Theme: hedonistic motives

Almost half of ethnic minorities (*n* = 8) rationalized their continued alcohol usage as a source of happiness, entertainment, relaxation, and self-satisfaction. They predominantly drank with their friends to unwind, and they eventually became addicted to it. For example, one participant mentioned during his interview, *“Um, just to chill with friends, and I do like alcohol. I did not like everyone, occasionally. Yeah, just like I do not know. OK, just saying to chill, just to feel good sometimes I needed”* (24-year-old male African).

#### 3.2.3 Theme: curiosity

Some participants (*n* = 5) drink alcohol to appreciate its effects and taste. A 17-year-old male participant stated that he drank alcohol to experience the negative effects of alcohol. During the interview, he said, *“Well, usually I’ve never drunk alcohol in my life, but I started it last year because I wanted to know, like, how it feels or what does it do to a man or what it does to a person, so yeah”* (17-year-old male, Pakistan). Four of the participants were curious about the taste of alcohol, and then they started drinking.

#### 3.2.4 Theme: coping motives

Almost half of the participants (*n* = 8) began and became addicted while drinking alcohol as a coping strategy for their negative emotions, like stress, depression, and anxiety. They drank to cope with stress related to work, school, or culture. Three participants were stressed because their school-grade success fell below their family’s expectations. For example, one participant stated in his interview, “*Because, as I said, there’s like stress from school. I feel like it’s a side we have never reached out to because my friend and I would have good terms with our parents, but we cannot. We do not have anyone to talk to. So, we do not know what to do, so you drink. We cannot, yeah”* (16-year-old male African). Another significant motive for their drinking was coping with difficult working conditions, low wages, unemployment, and lack of financial support from the government, which contributed to a stressful life. This, in turn, led them to use alcohol as a means of relief. For instance, one participant expressed during his interview, “*You know, maybe the house you are living in, rent is expensive to rent, the government is not paying for you. So, you must work hard to get there, and come to your family’s aid. Sometimes business stresses, you know. I know Hong Kong has so much stress; to be happy, raise yourself. You know, you come down; maybe you are working too much or like stressful thinking to make it come down, just to be yourself. Then, at least one hour or two hours of drinking”* (29-year-old male African).

#### 3.2.5 Theme: low-risk perceptions

One of the reasons that participants continued to drink was their low-risk perceptions of the detrimental impacts of AUD on their health. For instance, three participants believed that drinking was not inherently hazardous to their health. They mentioned that they needed to continue drinking as a part of their life unless medical indications were evident. For example, a participant stated, “*There’s nothing wrong with it. I mean, if you can handle it, then why not drink? If you cannot handle it, then you stop. There’s nothing wrong with alcohol. It depends on you. If your body can handle it, then drink if you can. If you are sick or something, then you should stop it, yeah”* (23-year-female, Filipino). One African participant unexpectedly noted in a semi-structured interview that red wine is essential and affordable. *He said, “Trust me, red wine is half worn, but all the people have great red wine; they do not call them alcoholics because they drink responsibly. So that’s what I’m going to do; I can drink the red one. That’s why I like to put my body in red wine, which is very good. Apart from beer, the prices were good”* (29-year-old male, African).

#### 3.2.6 Theme: social influences

Social influences were the major reasons for initiation and addiction to alcohol (*n* = 12). Peer pressure (*n* = 4) significantly contributed to initiating and maintaining drinking in different places and events, such as bars, restaurants, 7-Eleven shops, and other recreational areas. Most participants (*n* = 7) reported that they drank alcohol with friends every Sunday, on holidays, at birthday celebrations, and after tough work. For example, in the semi-structured interview, a participant said, *“It’s like when company friends go out and I taste the alcohol, I feel I can continue to drink. Then I feel excited. I mean myself. I teach me”* (25-year-old female, African). Another female participant also stated, *“Through friends. I was invited to a party and introduced to alcohol”* (24-year-old female, African).

#### 3.2.7 Theme: subjective cravings

Subjective cravings (*n* = 3) were the reasons for developing AUD. For instance, one participant stated that he felt bored, lonely, and lost when he tried to quit, and hence, he kept drinking, which further worsened his problem. In the semi-structured interview, he said, *“I found myself to be so lonely and so bored and so lost. That I was like, I cannot live like this. No social activity. I feel lost, I feel empty, with nothing to do. Yeah, no social activity. So, you know”* (29-year-male-African).

### 3.3 Impacts of AUD on ethnic minority young adults’ daily lives

#### 3.3.1 Theme: ineffective daily performance

AUD severely affected the work and activity of ethnic minority young adults. Half of the participants (*n* = 10) reported that they impaired their ability to focus on work, disrupted their sleep habits and mental well-being, caused feelings of lethargy, and made it more difficult to wake up early for work. For instance, a participant from Africa stated during his semi-structured interview, *“Well, if I have a hangover, I find it very difficult to wake up and go to work. I find it very hard to work because it gets annoying when people put pressure on you, and then your mental state is not OK. So, it affects my progress at work if I drank too much the previous night”* (29-year-old male, African).

Apart from their work, participants (*n* = 5) reported that AUD had an impact on their academic performance by decreasing their cognitive function, concentration, and sleeping patterns. For example, a participant from Pakistan said that AUD caused long-term memory loss, which had significantly impacted his academic ability. In his semi-structured interview, a participant stated, *“Yeah, it does kind of like, make me, like, you know, have long-term memory losses in learning, like, for example, if I do not know the formula or if I do not remember this, then it affects me because usually, like, I would learn everything, like, according to order. But if I forget about something, then that’s my problem at the end of the day”* (17-year-old male, Pakistan).

#### 3.3.2 Theme: emotional turmoil

Another important impact of AUD on ethnic minority young adults was emotional turmoil (*n* = 3). For instance, a female participant perceived herself as dying due to her feelings of desperation and helplessness in her day-to-day activities. She said in her semi-structured interview, *“If inside, I feel so dying, I feel if I get drunk like this, but if I am in that moment I’m drinking, I feel like I am high. This is the effect of this I am experiencing. But afterward, then I will feel like dying because of body pain. But this happened”* (27-year-female-Filipino).

#### 3.3.3 Theme: financial constraints

Participants’ budgets (*n* = 3) were considerably impacted by AUD. For example, one Filipino female participant indicated that her alcohol consumption had a significant influence on her budget because she always bought alcohol for herself as well as for her friends. As illustrated in her semi-structured interview, “*It affected my budget because sometimes my budget is for this one, and then if I go to my friend, like today, you see, I pay every day. For buying, I need a budget. That is, it”* (29-year-old female Filipino).

#### 3.3.4 Theme: social relationship snags

Because of AUD, some participants (*n* = 12) experienced communication and interaction challenges with their family and friends. They were embarrassed to tell their parents about drinks because they were afraid that their parents would not approve of their drinking. One of the female African participants stated that if her parents were open to her, she would be honest and open to them. As stated in her interview, “*One of my parents does not approve of me to drink. So only one of my parents knows. So, I’m being honest with another one because it’s being open with me. But another one does not like that I’m not honest with them. Because they are not open with me”* (19-year-old female, African).

Similarly, another two participants stated that they did not have an open conversation about their drinking habits and needed to hide alcohol from their parents. As stated in one of the interviews, *“It’s, you know, I feel ashamed because when I meet my relatives, I cannot show them what I’m doing. It’s a separate thing. I can do these things when they are not seeing them. I feel I give them respect. I cannot show these things because I know it’s bad. They disagree about what I’m doing, so I cannot show them”* (30-year-old female Filipino).

Moreover, participants (*n* = 5) had conflicts with friends because of their intake, such as misunderstanding, hitting, throwing up, injuring, and arguing. A participant from the Philippines stated in her interview that she lost control while she was drunk, and as a result, she beat people nearby. She stated in her semi-structured interview, *“When I get drunk, I’m more bubbling. Yeah, this has happened to me. Also, I have sometimes beaten someone. This I cannot control when I get drunk, so lucky to have my friends who cannot leave me if I get drunk because they know what will happen to me if I get what will happen to someone I can beat” (27-year-female, Filipino).*

## 4 Discussion

AUD is prevalent among ethnic minority young adults (40, 41). However, the literature on its impact on their daily lives and the reasons contributing to AUD was scarce. To address this gap in existing literature, the current study explored reasons for developing AUD and its impact on the daily lives of ethnic minorities.

Based on the qualitative interviews, we found seven reasons leading to AUD in ethnic minority young adults. Similar to previous literature (42–44), curiosity, hedonistic motives, social influence, and subjective craving were shown to be major reasons. Apart from these common reasons, our results revealed that the culture and family of the ethnic minority did significantly contribute to the increased prevalence of AUD in this population. Unlike people in Hong Kong, the ethnic minorities have their drinking traditions, such as sharing alcohol at different social events and drinking songs (45). For example, half of the participants in this study were Filipinos who were expected to be deeply influenced by their drinking traditions, in which drinking was considered an essential aspect of their lives because bars and pubs are common in their country. Some of our participants were African. They generally had a favorable attitude towards alcohol use. Particularly, they considered that alcohol was part of their life because their families approved of their drinking, and they enjoyed mass drinking with their family members in different social events as a celebration.

Apart from the cultural and family influence, relying on alcohol as a way of coping with negative emotions, especially acculturation stress, was also found to be a reason leading to AUD in ethics minority young adults. As revealed in the semi-structured interviews, ethnic minority young adults were facing a considerable degree of discrimination and various difficulties in adapting themselves to Hong Kong. Particularly, Hong Kong is a well-developed area dominated by Chinese (46). Most participants in this study reported that they were only offered opportunities for low-skilled jobs, notwithstanding that they were highly educated in their own countries. The participants who were students also encountered difficulties in reaching out to friends, as the Chinese were dominant in their schools (46). The difficulty in adapting to Hong Kong culture resulted in acculturation stress, and finally, some of our participants tended to evade their negative experiences and stress via drinking.

One more reason contributing to AUD in ethnic minority young adults in Hong Kong was the low-risk perception of alcohol use to their health. As illustrated in the semi-structured interviews, some participants understood the harmful effects of alcohol use. Nevertheless, they thought that through gradual alcohol use, their tolerance to alcohol would increase, and hence, drinking might not have a significant health impact on their health. Likewise, some participants said they were young and the serious health effects of alcohol would not be obvious to them. Therefore, they would continuously drink alcohol as long as there were no overt medical problems. Low-risk perceptions in young adults were similarly observed in other unhealthy behaviors, including smoking, physical inactivity, and unhealthy diet (47–53). A possible reason explaining their low-risk perceptions in multiple health risk behaviors, including AUD, could be due to their unique feelings of invulnerability, as they usually consider that the health consequences are too remote. Another specific reason accounting for the low health risk perception in ethnic minority young adults was that some of these adults were from low-income countries with relatively low educational levels (54). Hence, they might not have an adequate understanding of the health consequences of AUD. Ironically, although some diseases associated with AUD may only become obvious in older ages, compelling evidence has shown that alcohol drinking, even for periodic involvement, can result in numerous health consequences for young adults (55, 56). This finding indicates the importance of clarifying the misconceptions of ethnic minority young adults towards AUD, thus heightening their risk perception.

This study also explored how AUD affected the daily lives of ethnic minority young adults. Our results found that the impact could be classified into four aspects, including ineffective daily performance, financial constraints, emotional turmoil, and relationship snags. For the ineffective daily performance, most participants admitted that their work and academic performance were greatly impaired due to the hangover effects of alcohol, such as weakness, headache, fatigue, and vertigo, resulting in late school or work and reduced concentration on academic or work activities. For financial constraints, some participants who had AUD reported that they had spent a large amount of their income on alcohol, leaving them with an insufficient budget for other activities, including essential activities. These qualitative findings indeed reflect a paradoxical phenomenon. On one hand, drinking was regarded by some ethnic minority young adults as a way of coping with and managing negative emotions and stress in acculturation (57, 58). On the other hand, constant drinking of alcohol resulted in addiction, limiting the physical functions and budget to engage in daily activities, which in turn increases more obstacles and stress for ethnic minority young adults to adapt to Hong Kong (59). The struggles in this paradoxical phenomenon among ethnic minority young adults with AUD were also reflected in the third aspect of the impact, which is emotional turmoil. While the participants were tipsy or drunk, they felt high as they were emotionally relieved from stress and negative emotions. However, when the participants became conscious, they were helpless and felt hopeless about their lives and future. Despite these ethnic minority young adults being trapped in this vicious cycle, they found it difficult to seek help from parents and friends, especially if their parents and friends were not open to discussing alcohol use. As shown in the fourth dimension of the impact, which is relationship snags, some participants did not inform their parents about their AUD because drinking was not approved by their parents. In addition, some participants reported that their relationship with friends was seriously affected by their behaviors, e.g., violent attacks after getting drunk. In sum, based on the qualitative interviews, it was observed that ethnic minority young adults with AUD struggled with alcohol as a way of coping with the acculturation stress as well as the physical, emotional, social, and financial impacts of AUD on their lives. Unlike other diseases, these ethnic minority young adults could only receive minimal support from parents and friends for AUD due to non-disclosure and/or ruined social relationships.

## 5 Implications for future research

Based on the findings, some recommendations are provided for additional investigation. Firstly, one of the key reasons for developing AUD is coping motives. Some ethnic minority young adults drink alcohol as a coping technique for their internal and external unpleasant experiences, such as stress, depression, anxiety, mood change, and a fear of social isolation. Although some know the consequences of getting drunk, they think drinking is necessary for them to cope with stress. This experience-avoidant behavior remains a ground for AUD, and this evidence confirms the need for researchers to develop and implement appropriate interventions to assist coping in ethnic minority young adults, especially when compared to the locals, who are exposed to additional stress from acculturation. A review of the literature revealed that acceptance and commitment therapy, as the 3rd wave of behavior therapies, may be an appropriate and feasible treatment option (60–62). As reflected in our qualitative interviews, relying on drinking to cope with stress and negative emotions from acculturation led to more obstacles and discrimination in their lives, resulting in more stress and negative emotions for ethnic minority young adults, which in turn makes them further apart from their goal, which is integrating themselves into Hong Kong society. Acceptance and commitment therapy is an action-oriented approach to assist clients in accepting instead of avoiding and struggling with their negative feelings and responses and committing to behaviors to achieve their life goals (63). Given the nature of acceptance and commitment therapy, this treatment approach appears to be suitable to address the needs of ethnic minority young adults with AUD and warrants further examination in future trials. Secondly, despite this qualitative study identifying different factors leading to the development of AUD in ethnic minority young adults, more quantitative research needs to be conducted to clarify the statistical connections of these factors with AUD, thus facilitating the intervention development.

## 6 Limitations

Despite the originality of the current investigation, numerous limitations have been observed. Firstly, we employed the snowball sampling technique to recruit participants by referrals from friends, and most participants suggested others from their nation, which decreased the number of participants to the three ethnic groups only: Filipino, African, and Pakistani. As a result, the findings might not be transferable to other ethnic minority groups. Secondly, a majority of our participants were female, attributed to the fact that most were coming to Hong Kong as domestic helpers, which is a female-dominant job (64, 65). Hence, this further limited the transferability of our findings, particularly to males. Third, only individuals who could speak, hear, read, and comprehend English were interviewed for this study, potentially introducing selection bias. Fourth, all participants in this study were recruited in the community; consequently, the diagnosis of AUD exclusively relied on the participants’ self-reported drinking status.

## 7 Conclusion

This study showed the complex interplay of factors contributing to AUD among ethnic minority young adults, encompassing curiosity, social influences, coping strategies, and cultural and family contexts. The detrimental effects of AUD on emotional health, financial stability, and interpersonal relationships highlight the urgent need for tailored interventions. To effectively address the unique challenges faced by this demographic, further quantitative research is imperative, paving the way for the development of culturally appropriate strategies.

## Data Availability

The raw data supporting the conclusions of this article will be made available by the authors, without undue reservation.
