# Supplementary material for: Reasons and impacts of alcohol use disorder among ethnic minority young adults: a descriptive phenomenological study
Source: Front Public Health. 2025 Jun 19;13:1560092. doi: 10.3389/fpubh.2025.1560092 (PMC12222166; doi:10.3389/fpubh.2025.1560092)
Supplement: Supplementary file 1 [file Data_Sheet_1.docx]

**Supplementary file 1:** Interview guide questions

1. **Descriptions and reasons for alcohol use disorders**

- How are you feeling today?
- Let’s talk about your drinking experiences. Is there anything that probably leads you to drink in your day-to-day activities? What happened to you?
- How did you start drinking? Why do you drink?
- For how long have you lived with AUD?
- Could you explain more about your AUD?
- Tell me about the reasons for how you develop AUD

1. **How AUD affects your life**

- Let’s talk about the impact of AUD on your life.
- How has AUD affected your daily life?
- Can you explain more about your addiction effect on your learning or works?
- Do your parents know about your drinks? What is your relationship with family?
- What is your social interaction with friends and neighbors?
- Tell me about any changes in your behavior due to drinking? What happened?
- What problems or consequences are you experiencing because of your addiction?
