# Supplementary material for: Reasons and impacts of alcohol use disorder among ethnic minority young adults: a descriptive phenomenological study
Source: Front Public Health. 2025 Jun 19;13:1560092. doi: 10.3389/fpubh.2025.1560092 (PMC12222166; doi:10.3389/fpubh.2025.1560092)
Supplement: Supplementary file 2 [file Table_1.docx]

**Supplementary Table 1:** Bracketing process

| Stages | Actions |
| --- | --- |
| Pre-Interview | We identified assumptions related to AUD in ethnic minorities   - Discrimination causes AUD - Culture significantly contributed to AUD - Acculturation stress leads to AUD |
| Interview | - Use open-ended questions - E.g. - Why do you drink alcohol? - Tell me about the reasons for developing AUD   -Field note taking   - Record interview setting, date, time, and nonverbal clues - Observation of emotional reactivity |
| During analysis | - Two researchers independently coded transcripts - Disagreement resolved through discussion |
| Post-study | - Checking over process - Team debriefs to discuss developed themes - Comparing findings with pre-assumptions |
